# Experiences of harm and mental ill-health among gay, bisexual and other men-who-have-sex-with-men who use methamphetamine or GHB/GBL in different combinations: findings from the COMeT study in Taiwan

**DOI:** 10.1186/s12954-024-01094-8

**Published:** 2024-10-07

**Authors:** Jing-Hao Hsu, Poyao Huang, Chia-Wen Li, Adam Bourne, Carol Strong, Stephane Wen-Wei Ku

**Affiliations:** 1 Department of Public Health, College of Medicine, National Cheng Kung University Hospital, National Cheng Kung University, 8F-8068, No. 138, ShengLi Rd., North Dist., Tainan City, 704 Taiwan; 2 Institute of Health Behaviors and Community Sciences, College of Public Health, National Taiwan University, Taipei, Taiwan; 3 Master of Public Health Degree Program, College of Public Health, National Taiwan University, Taipei, Taiwan; 4 Division of Infectious Diseases, Department of Internal Medicine, National Cheng Kung University Hospital, College of Medicine, National Cheng Kung University, Tainan, Taiwan; 5 Australian Research Centre in Sex, Health and Society, La Trobe University, Melbourne, Australia; 6 Kirby Institute, UNSW Sydney, Sydney, Australia; 7 Division of Infectious Diseases, Department of Medicine, Taipei City Hospital Renai Branch, Taipei, Taiwan

**Keywords:** Gay-bisexual-and-other-men-who-have-sex-with-men (GBMSM), Chemsex, Methamphetamine, Gamma-hydroxybutyric acid (GHB), Gamma-butyrolactone (GBL), Harm reduction

## Abstract

**Introduction:**

Polydrug use in the context of chemsex is commonplace among gay, bisexual, and other men-who-have-sex-with-men (GBMSM). This study aimed to examine the differences in experiences of physical, social, and psychological harms, as well as mental ill-health among GBMSM who use different combinations of methamphetamine and gamma-hydroxybutyric acid/gamma-butyrolactone (GHB/GBL) during chemsex.

**Method:**

Adult GBMSM participants who had experience of chemsex in the past 12 months participated in a cross-sectional online survey in Taiwan and self-reported their sociodemographic background, sexual behaviours, mental health, and experiences of harm following a chemsex session. We used univariable and multivariable logistic regression to assess the different experiences of harm and mental ill-health among GBMSM who engaged in chemsex without using methamphetamine, used methamphetamine but not GHB/GBL, and who used both drugs.

**Results:**

Out of 510 participants who completed all items included in the analysis, 24.1% engaged in chemsex without using methamphetamine, 36.9% used methamphetamine but not GHB/GBL, and 39.0% used both drugs. Eighty five percent of men who used both methamphetamine and GHB/GBL reported at least one kind of social harm after a chemsex session, such as missing dates or appointments, or appearing “high” at work, followed by used methamphetamine but not GHB/GBL (69.7%) and those without using methamphetamine (37.4%). After controlling for polydrug and frequency of drug use in the multivariable logistic regression, those who used methamphetamine but not GHB/GBL and those who used both drugs were more likely to report experiencing physical and psychological harms compared to those who did not use methamphetamine (*p* < 0.003).

**Conclusion:**

GBMSM who used both methamphetamine and GHB/GBL in a chemsex context were more likely to report experience of harms than those who only used a single chemsex drug or engaged in chemsex without methamphetamine or GHB/GBL. Harm reduction should focus on both preventing HIV and STI transmission and on minimising psychosocial harm to GBMSM, with varying impacts depending on drug use.

## Introduction

Chemsex is a form of sexualized drug use, broadly defined by the use of methamphetamine, gamma-hydroxybutyric acid/gamma-butyrolactone (GHB/GBL), or mephedrone during or before sexual activities to enhance sexual pleasure or performance in gay, bisexual, and other men-who-have-sex-with-men (GBMSM) [1]. It has emerged as an issue of public health concern that spans both psychosocial issues as well as those issues relating to transmission of the human immunodeficiency virus (HIV) and other sexually transmitted infections (STIs) among this population [2]. Chemsex is not intrinsically problematic and research has documented careful, controlled engagement without adverse outcomes [3, 4], and has highlighted the role drugs can play in advancing sexual pleasure [5]. However, chemsex-related harm, such as drug overdose or substance dependency [4, 6], has also been documented, as have negative impacts on pre-exposure prophylaxis (PrEP) adherence or reduced mental health [7–9]. Moreover, some studies have indicated that GBMSM who engage in sexualized drug use can sometimes report lower life satisfaction or lower sexual self-efficacy [10]. Chemsex, as a subset of sexualized drug use, has also been associated with a higher risk of depression and substance use disorders compared to those who do not use drugs in this manner [11, 12]. Caution is needed when interpreting these negative associations between chemsex and well-being due to limitations in the predominantly cross-sectional study design, which requires further exploration.

Most studies have taken a broad approach to examining chemsex practices, often analyzing drug use as a single group without adequately exploring individual substances or focusing solely on polydrug use without examining potential synergistic interactions. Some studies have focused on only a single chemsex drug, such as methamphetamine or GHB/GBL [13, 14], and rarely have studies assessed the potential synergistic effect of mixed drug use, despite polydrug use being widely reported among this population and in this context [15, 16]. Two drugs that appear to be commonly featured as part of chemsex, often (but not always) used in combination [17], are methamphetamine and GHB/GBL, as seen in England [18], Germany [19], Australia [20], and Taiwan [6]. In Taiwan, nearly 88% of GBMSM respondents to a large online chemsex survey had used methamphetamine and 48% had used GHB/GBL in the last six months [6].

Methamphetamine and GHB/GBL act differently upon the central nervous system (CNS) and can be associated with different harms, possibly due to their pharmacological properties [21]. Methamphetamine is a CNS stimulant that often induces raised heart rate, loss of appetite, and sweating [22]. On the other hand, GHB/GBL, a popular drug in the party scene in GBMSM since the 1990s [23, 24], is a CNS depressant that can result in respiratory depression and bradycardia [25]. In addition to the physical effects, methamphetamine and GHB/GBL can be associated with mental health outcomes (e.g., mood-related problems) as well as harms to social well-being, such as interference in daily activities. For example, GHB/GBL can easily result in overdose when used in combination with other drugs [26]. The increase of the frequency and the time of using GHB/GBL may lead to drug dependence and withdrawal symptoms [27]. These properties might impair the user's social function and engagement in daily activities (such as impacts on employment and absenteeism) [26–28]. Methamphetamine can impair a user’s cognitive function [29], heightening the risk of social consequences such as family or financial problems [30]. Despite these differences in effect, we are not aware of any published research to date that has examined differential health and social outcomes among GBMSM who use GHB/GBL or methamphetamine in isolation or use them both.

Given the different characteristics of methamphetamine and GHB/GBL, as outlined above, it is necessary to separately examine the association of these two main drugs of chemsex with mental health and harms. This paper examines the following: (1) differences in the sociodemographic characteristics, sexual behaviours, and reported STI diagnoses, and (2) differences in physical, social, and psychological experiences of harm associated with chemsex for GBMSM who use GHB/GBL and/or methamphetamine.

## Materials and methods

### Study design, setting, and participants

The Chemsex Online Survey for Men who Have Sex with Men in Taiwan (COMeT) was an anonymous cross-sectional online study that investigated the characteristics of harm in chemsex scenarios among GBMSM in Taiwan [6]. The survey was advertised on smartphones through a targeted GBMSM social networking application. Participants were recruited when they met the following criteria: (1) male aged 20 years or above, (2) had an experience of sex with men, (3) lived in Taiwan, and (4) had used the following drugs during a sexual context in the past 12 months: methamphetamine, ketamine, 3,4-Methylenedioxymethamphetamine (MDMA), or GHB/GBL. All data were collected between December 2018 to January 2019. The study was approved by the Institutional Review Board of National Cheng Kung University Hospital (A-ER-107–329).

### Variables

#### Methamphetamine and/or GHB/GBL use

All participants reported whether they had engaged in chemsex or sexualized-related drug use in the past 6 months with any of the following substances: MDMA, prescribed sedative drugs (such as Stilnox), amyl nitrite, erectile dysfunction (ED) drugs, ketamine, methamphetamine, 5-methoxy-diisopropyltryptamine (5-meo), alcohol, marijuana, mephedrone, GHB/GBL, cocaine, and “coffee-pack” (a pack of mixed substances with unknown ingredients). Participants were originally categorised into four groups based on their use (+) or not use (−) of methamphetamine (M) and/or GHB/GBL (G) in the past 6 months: M−G−, M−G+, M+G− and M+G+. Given that the proportion of M﻿−G+ participants (*n* = 20) was less than 5% in the data of final analysis, we then combined the M−G+ and M−G− as the group who did not use methamphetamine (M−).

#### Other substance use variables

Polydrug use was defined as the use of drugs excluding methamphetamine, GHB/GBL, alcohol, and ED drugs from the list of drugs in the survey. Methamphetamine and GHB/GBL were excluded due to multicollinearity. The frequency of using methamphetamine or GHB/GBL was assessed in separate questions by asking how often they used methamphetamine or GHB/GBL on a nine-point Likert scale from “daily” to “never.”

#### Sociodemographic characteristics

We included age (20–29, 30–39, and > 39 years old), education (college or below, college graduate, and above college), and monthly income (< 15 kilos [K] New Taiwan dollars [NTD], 15–30 K NTD, and > 30 K NTD; 1 United States dollar [USD] ≅ 30 NTD).

#### Sexual behavior

We asked each participant to report the number of sexual partners they had in the previous 12 months and categorised them as < 3, 3–5, 6–9, or > 9 partners. We also asked about their frequency of using condoms in a chemsex context in the past three months and categorised them as inconsistent (i.e., not always) and consistent (i.e., always).

#### HIV status and STI diagnoses

We combined the self-reported status of HIV and PrEP uptake in the past three months in a single variable and categorised it as HIV negative on PrEP, HIV negative but not on PrEP, and HIV positive. Self-reported STIs in the past 12 months, including gonorrhea, syphilis, anal and penile warts, genital herpes, amoebic colitis, chlamydia, shigellosis, and hepatitis virus infection (including type A, B, and C virus) were categorised into none and any.

#### Harm experienced after a chemsex session

All participants were asked whether they had experienced a range of issues following a chemsex session and were presented with a 17-item checklist. Items were categorised into physical (such as eating problems or missing PrEP or HIV drugs), social (such as having difficulty getting out of bed or absence from work, etc.), and psychological (such as auditory hallucination or paranoid, etc.) experiences.

#### Mental health

We included four indicators of mental health: mood-related problems, suicide ideation, loneliness, and sexual well-being. Mood-related problems was measured by the Taiwan version of the 5-item Brief Symptom Rating Scale (BSRS-5, Cronbach’s alpha: 0.77–0.90) on a scale of 0–4 [31]. Total scores that were 6 or above were categorized as having mood-related problems [31]. Suicidal ideation was defined by reporting other than “not at all” on the question of how much they were troubled by having suicidal thoughts during the past week. Loneliness was defined by reporting anything other than “none” on the question of how often they felt lonely in the past 3 months. Sexual well-being was assessed by asking how happy they were with their sex life in general on a five-point Likert scale from very unhappy (1) to very happy (5). Sexual well-being was defined as those who reported more than 2.

### Statistical analysis

We conducted chi-square tests for descriptive statistics (including frequencies and proportions) to compare different mixed drugs groups (M−, M+G−, and M+G+) on sociodemographic characteristics, sexual behaviour, the status of STIs, experiences of harm after a chemsex session and mental health. Univariable and multivariable logistic regression was used to assess the associations among different mixed drug groups and experiences of harm after a chemsex session. We set the alpha level at 0.05 and adjusted the alpha level for the multiple comparisons by using Bonferroni’s method (altered alpha level = original alpha divided by the number of dependent variables). All data were analysed by Intercooled STATA software version 15.0 (College Station, TX).

## Results

### Sociodemographic characteristics

A total of 918 participants met the eligibility criteria for inclusion in the online survey, we deleted duplicated answers (*n* = 13), only provided consent but did not start the survey (*n* = 204), less than 20 years old (*n* = 34), did not engage in chemsex in the past six months (*n* = 737). We kept only persons with complete answers for all the covariates, which left 510 participants for the purposes of this paper. Table 1 reports on their sociodemographic characteristics, sexual behaviour, and the status of HIV and STIs of participants, broken down by whether they were among the M﻿− (*n* = 123, 24.1%), M+G﻿− (*n* = 188, 36.9%), and M+G+ (*n* = 199, 39.0%) profile groups. The three groups showed no significant difference in terms of age, education, or income.

**Table 1:** The sociodemographic characteristics, sexual behaviour, HIV status, and STIs among different GBMSM chemsex groups

	Total *N* (%) *N* = 510	M− *n* (%) 123 (24.1)	M+G− *n* (%) 188 (36.9)	M+G+ *n* (%) 199 (39.0)	*χ*^*2*^	*p*-value
Sociodemographic characteristics						
Age (years old)					6.98	0.137
20–29	175 (34.3)	34 (27.6)	68 (36.2)	73 (36.7)		
30–39	246 (48.2)	59 (48.0)	89 (47.3)	98 (49.3)		
> 39	89 (17.5)	30 (24.4)	31 (16.5)	28 (14.1)		
Education					3.98	0.409
College or below	85 (16.7)	18 (14.6)	35 (18.6)	32 (16.1)		
College graduate	333 (65.3)	79 (64.2)	127 (67.6)	127 (63.8)		
Above college	92 (18.0)	26 (21.1)	26 (13.8)	40 (20.1)		
Income (NTD per month)					6.79	0.147
< $15 K	55 (10.8)	12 (9.8)	17 (9.0)	26 (13.1)		
$15–$30 K	142 (27.8)	31 (25.2)	64 (34.0)	47 (23.6)		
> $30 K	313 (61.4)	80 (65.0)	107 (56.9)	126 (63.3)		
Sexual behaviour						
Number of sexual partners in the past 12 months					34.12	< 0.001^***^
< 3	92 (18.0)	34 (27.6)	45 (23.9)	13 (6.5)		
3–5	125 (24.5)	30 (24.4)	47 (25.0)	48 (24.1)		
6–9	106 (20.8)	25 (20.3)	35 (18.6)	46 (23.1)		
> 9	187 (36.7)	34 (27.6)	61 (32.5)	92 (46.2)		
Condom use during chemsex in the past 3 months					52.99	< 0.001^***^
Inconsistent	429 (84.1)	79 (64.2)	162 (86.2)	188 (94.5)		
Consistent	81 (15.9)	44 (35.8)	26 (13.8)	11 (5.5)		
HIV and STIs						
HIV status and PrEP uptake					53.47	< 0.001^***^
HIV negative, on PrEP	61 (12.0)	15 (12.2)	16 (8.5)	30 (15.1)		
HIV negative, not on PrEP	194 (38.0)	76 (61.8)	72 (38.3)	46 (23.1)		
HIV positive	255 (50.0)	32 (26.0)	100 (53.2)	123 (61.8)		
STIs^a^ (excluding HIV infection)					44.78	< 0.001^***^
Never	282 (55.3)	97 (78.9)	104 (55.3)	81 (40.7)		
Any STIs	228 (44.7)	26 (21.1)	84 (44.7)	118 (59.3)		

HIV, human immunodeficiency virus; STIs, sexually transmitted infections; GBMSM, gay, bisexual, and other men-who-have-sex-with-men; M−, chemsex individuals who did not use methamphetamine in the past six months; M+G−, chemsex individuals who use methamphetamine but not GHB/GBL in the past six months; M+G+, chemsex individuals who used both methamphetamine and GHB/GBL in the past six months; NTD, New Taiwan Dollar; K, kilo dollar; PrEP, pre-exposure prophylaxis

^a^Self-reported diagnosis of sexually transmitted infections in the preceding 12 months was reported including gonorrhea, syphilis, anal and penile warts, genital herpes, amoebic colitis, chlamydia, shigellosis, and hepatitis virus infection (including type A, B, and C virus)

^*^p < 0.05; ^**^p < 0.01; ^***^p < 0.001

### Sexual behaviour, the status of HIV and STIs

The three groups showed a significant difference in sexual behaviour and in terms of reported HIV status or experience of STI diagnosis (*p* < 0.001, Table 1). Over a third of participants (36.7%) reported that they had sex with ten or more sexual partners in the past 12 months (M+G+: 46.2%, M+G−: 32.5%, and M−: 27.6%). A total of 84.1% of participants used condoms inconsistently during chemsex in the past 3 months, which again varied by group (M+G+: 94.5%, M+G−: 86.2%, and M﻿−: 64.2%).

Half of the participants were living with diagnosed HIV (50.0%), 12% were HIV negative on PrEP, and 38% were not on PrEP. The M+G+ group included the highest proportion of people living with HIV (61.8% compared with 26.0% in M﻿− and 53.2% in M+G−) and the highest proportion on PrEP (15.1% compared with 12.2% in M− and 8.5% in M+G−). Nearly half of the participants (44.7%) had experienced at least one STI diagnosis in the last 12 months, excluding HIV (M+G+: 59.3%, M+G−: 44.7%, and M﻿−: 21.1%).

### Harm experienced after a chemsex session

Tables 2 and 3 respectively present comparisons of the proportion, odds ratio (OR), and 95% confidence interval (95% CI) in harm experienced after a chemsex session, as well as the mental health profiles of the three different groups. After adopting Bonferroni's altered alpha for multiple comparisons (altered alpha, *α*_*alter*_ = 0.05/21 = 0.00238), Table 2 displays results that show significant differences in the proportion of all physical and psychological harm experienced among the three groups, including problems eating (M+G+: 70.9%, M+G−: 54.8%, and M−: 30.1%) and sleeping (M+G+: 78.9%, M+G−: 67.6%, and M−: 37.4%). With respect to almost all post-chemsex session harms, there were significant differences between the three groups, including absence from work (M+G+: 33.7%, M+G−: 21.3%, and M-: 7.3%) and missing dates or appointments (M+G+: 44.2%, M+G−: 23.4%, and M−: 9.8%). No differences were found in items such as being verbally offensive or physical assaults on others, being unable to remember what happened, and trading sex for drugs (see Table 2). The highest reports of physical, psychological, and social harm were reported among the M+G+ group, followed by M+G− and M−. Being verbally offensive or physically assaulting others were the only two experiences where the M+G− group reported a higher proportion than the M+G+ group, although the differences were not significant.

**Table 2:** The comparisons of harm experiences and mental health among different GBMSM chemsex groups

Variable	Total		M−		M+G−		M+G+		*χ*^*2*^	*p*-value
	*N*	%	*n*	%	*n*	%	*n*	%		
Harm experienced after a chemsex session										
*Physical experiences*										
Eating problems	281	55.1	37	30.1	103	54.8	141	70.9	51.09	< 0.001^*^
Missing PrEP or HIV drugs	84	16.5	7	5.7	33	17.6	44	22.1	15.15	0.001^*^
* Any physical experiences*	298	58.4	37	30.1	111	59.0	150	75.4	64.26	< 0.001^*^
*Social experiences*										
Having difficulty getting out of Bed or standing up	114	22.4	15	12.2	38	20.2	61	30.7	15.71	< 0.001^*^
Absence from work	116	22.8	9	7.3	40	21.3	67	33.7	30.40	< 0.001^*^
Missing dates or appointments	144	28.2	12	9.8	44	23.4	88	44.2	47.99	< 0.001^*^
Being verbally offensive to others	56	11.0	5	4.1	26	13.8	25	12.6	8.09	0.018
Having physical assaults on others	8	1.6	0	0.0	5	2.7	3	1.5	3.41	0.181
Being unable to remember what Happened	53	10.4	8	6.5	20	10.6	25	12.6	3.02	0.221
Spending more money on drugs than as intended	107	21.0	10	8.13	39	20.7	58	29.2	20.26	< 0.001^*^
Spending more time with drugs and sex than as intended	209	41.0	23	18.7	73	38.8	113	56.8	46.16	< 0.001^*^
Trading sex for drugs	56	11.0	4	3.3	23	12.2	29	14.6	10.45	0.005
Appearing “High” at work	146	28.6	16	13.0	54	28.7	76	38.2	23.60	< 0.001^*^
* Any social experiences*	346	67.8	46	37.4	131	69.7	169	84.9	79.16	< 0.001^*^
*Psychological experiences*										
Unstable mood	185	36.3	29	23.6	64	34.0	92	46.2	17.52	< 0.001^*^
Anxiety	197	38.6	29	23.6	69	36.7	99	49.8	22.43	< 0.001^*^
Auditory hallucination	91	17.8	8	6.5	34	18.1	49	24.6	17.04	< 0.001^*^
Paranoid	118	23.1	9	7.3	49	26.1	60	30.2	23.72	< 0.001^*^
Sleep problem	330	64.7	46	37.4	127	67.6	157	78.9	58.37	< 0.001^*^
* Any psychological experiences*	382	74.9	55	44.7	147	78.2	180	90.5	86.30	< 0.001^*^
Mental health indicators										
Mood-related problems	190	37.3	33	26.8	66	35.1	91	45.7	12.20	0.002^*^
Suicide ideation	141	27.7	20	16.3	51	27.1	70	35.4	13.86	0.001^*^
Loneliness	487	95.5	112	91.1	182	96.8	193	97.0	7.40	0.025
Sexual well-being	408	80.6	101	82.8	145	78.4	162	81.4	1.04	0.594

GBMSM, gay, bisexual, and other men-who-have-sex-with-men; M−, chemsex individuals who did not use methamphetamine in the past six months; M+G− chemsex individuals who use methamphetamine but not GHB/GBL in the past six months; M+G+, chemsex individuals who used both methamphetamine and GHB/GBL in the past six months; PrEP, pre-exposure prophylaxis; HIV, human immunodeficiency virus

*p < 0.002 (Bonferroni's adjusted alpha level for multiple comparisons *α*_*alter*_ = 0.05/21 = 0.00238)

**Table 3:** Logistic regression analysis on harm experiences and mental health among different GBMSM chemsex groups

	Univariable		Multivariate multivariable					
			Model 1^a^		Model 2^b^		Model 3^c^	
	OR (95% CI)	*p-*value	OR (95% CI)	*p*-value	OR (95% CI)	*p*-value	OR (95% CI)	*p*-value
Harm experienced after a chemsex session								
Any physical experiences								
M+G− versus M−	3.35 (2.07–5.43)	< 0.001^*^	2.85 (1.72–4.73)	< 0.001^*^	3.05 (1.83–5.08)	< 0.001^*^	2.75 (1.47–5.15)	0.002^*^
M+G+ versus M−	7.12 (4.30–11.76)	< 0.001^*^	6.05 (3.50–10.45)	< 0.001^*^	5.67 (3.27–9.83)	< 0.001^*^	5.85 (2.94–11.64)	< 0.001^*^
Any social experiences								
M+G− versus M−	3.85 (2.38–6.22)	< 0.001^*^	3.35 (2.02–5.54)	< 0.001^*^	3.52 (2.11–5.87)	< 0.001^*^	2.09 (1.12–3.90)	0.021
M+G+ versus M−	9.43 (5.53–16.07)	< 0.001^*^	7.29 (4.12–12.91)	< 0.001^*^	6.91 (3.88–12.29)	< 0.001^*^	4.11 (2.04–8.27)	< 0.001^*^
Any psychological experiences								
M+G− versus M−	4.43 (2.70–7.28)	< 0.001^*^	3.94 (2.34–6.65)	< 0.001^*^	4.23 (2.48–7.22)	< 0.001^*^	3.51 (1.80–6.85)	< 0.001^*^
M+G+ versus M−	11.71 (6.48–21.16)	< 0.001^*^	9.15 (4.87–17.21)	< 0.001^*^	8.61 (4.56–16.26)	< 0.001^*^	6.55 (3.00–14.33)	< 0.001^*^
Mental health indicators								
Mood−related problems								
M+G− versus M−	1.48 (0.90–2.43)	0.126	1.29 (0.75–2.23)	0.358	1.30 (0.75–2.24)	0.353	0.89 (0.45–1.77)	0.737
M+G+ versus M−	2.30 (1.41–3.74)	0.001^*^	1.80 (1.03–3.14)	0.038	1.79 (1.02–3.14)	0.042	0.97 (0.47–2.00)	0.941
Suicide ideation								
M+G− versus M−	1.92 (1.08–3.41)	0.027	1.75 (0.95–3.24)	0.075	1.80 (0.97–3.33)	0.064	1.50 (0.72–3.16)	0.282
M+G+ versus M−	2.82 (1.61–4.93)	< 0.001^*^	2.40 (1.28–4.50)	0.006^*^	2.30 (1.22–4.35)	0.010	1.91 (0.87–4.16)	0.106
Loneliness								
M+G− versus M−	2.98 (1.07–8.28)	0.036	2.21 (0.75–6.49)	0.148	1.76 (0.58–5.34)	0.315	0.80 (0.22–2.94)	0.742
M+G+ versus M−	3.16 (1.14–8.78)	0.027	1.55 (0.49–4.91)	0.457	2.25 (0.65–7.82)	0.201	1.01 (0.23–4.49)	0.991
Sexual well−being								
M+G− versus M−	0.75 (0.42–1.35)	0.344	0.88 (0.47–1.63)	0.675	0.95 (0.51–1.78)	0.866	1.14 (0.52–2.46)	0.746
M+G+ versus M−	0.91 (0.50–1.64)	0.755	1.02 (0.53–1.98)	0.948	0.89 (0.45–1.74)	0.734	0.78 (0.34–1.80)	0.558

GBMSM, gay, bisexual, and other men-who-have-sex-with-men; OR, odds ratio; CI, confidence interval; M﻿−, chemsex individuals who did not use methamphetamine in the past six months; M+G﻿−, chemsex individuals who use methamphetamine but not GHB/GBL in the past six months; M+G+, chemsex individuals who used both methamphetamine and GHB/GBL in the past six months

^a^After controlling for age, income, number of partners, and infection with human immunodeficiency virus or sexually transmitted infections

^b^After controlling for variables which were controlled for in Model 1 and polydrug use excluding methamphetamine nor GHB/GBL

^c^After controlling for variables which were controlled for in Model 2 and frequency of using methamphetamine and GHB/GBL

*p < 0.007 (Bonferroni's adjusted alpha level for multiple comparisons *α*_*alter*_ = 0.05/7 = 0.00714)

In multivariable analysis (Table 3, *α*_*alter*_ = 0.05/7 = 0.00714), Model 1 showed that GBMSM in the M+G+ group were significantly more likely to report harms following a chemsex session compared to M﻿− (OR[95% CI] 6.05[3.50–10.45] for physical, 7.29[4.12–12.91] for social, and 9.15[4.87–17.21] for psychological experiences). GBMSM in the M+G− group were also significantly more likely to report harm compared to those in the M﻿− group (OR[95% CI] 2.85[1.72–4.73] for physical, 3.35[2.02–5.54] for social, and 3.94[2.34–6.65] for psychological experiences). Similar findings were found in Model 2 which controlled for variables that were controlled for in Model 1 and polydrug. Model 3 further controlled for the frequency of using methamphetamine and GHB/GBL showed that GBMSM in the M+G+ group were significantly more likely to report harms following a chemsex session compared to M− (OR[95% CI] 5.85[2.94–11.64] for physical, 4.11[2.04–8.27] for social, and 6.55[3.00–14.33] for psychological experiences). However, GBMSM in the M+G− group were only significantly more likely to report physical (OR[95% CI] 2.75[1.47–5.15]) or psychological (OR[95% CI] 3.51[1.80–6.85]) harm experiences compared to those in the M﻿− group.

### Mental health

Loneliness and sexual well-being showed no difference among the three groups. Even though suicidal ideation and mood-related problems were significant in univariable analysis comparing M+G+ and M−, they were not significant in Models 2 and 3 when polydrug use and frequency were further adjusted (see Table 3).

## Discussion

To the best of our knowledge, this is the first paper to distinguish and compare the profile of harm and mental health among GBMSM who engage in mixed patterns of chemsex drug use (i.e., methamphetamine and/or GHB/GBL). The users of methamphetamine and/or GHB/GBL showed no difference in their sociodemographic characteristics but there were differences in sexual behaviour, HIV status, and the recent diagnosis of an STI. Even after controlling for polydrug, frequency of drug use, and other potentially confounding variables, those who used methamphetamine but not GHB/GBL and those who used both drugs were more likely to report experiencing different categories of harm compared to those who did not use methamphetamine. Mental health, loneliness, suicidal ideation and sexual well-being were not different among the three drug combination groups in multivariable analysis. GBMSM in the M+G+ group were most likely to report almost all kinds of harm following a chemsex session. Moreover, the risk of experiencing every kind of harm followed a decreasing gradient from GBMSM in the M+G+, M+G−, and M− groups.

Our findings revealed that people who used both methamphetamine and GHB/GBL were most likely to report nearly every kind of harm after a chemsex session. Studies have found that the simultaneous use of drugs with opposite mechanisms (i.e., using CNS stimulant with CNS depressant) was associated with more toxic drug interactions than using a single drug, such as using ketamine with caffeine, using cocaine with alcohol, or using cocaine with heroin (also known as “Speedball”) [32–34]. However, it may also be the case that two drugs with opposite mechanisms might offset each other's adverse side effects and help people to overcome the disadvantages of drugs. For example, using methamphetamine might lead to sleep disturbance [35]. However, using GHB/GBL could reduce sleep-related problems [36].

It is worth noting that we found significant differences between M+G+ and M− in each type of harm after we further controlled for polydrug and frequency of using drug. This may reflect that beyond the effects of polydrug and frequency, the pattern of using drugs (i.e., using both methamphetamine and GHB/GBL) itself was still one of the important factors that was associated with different kinds of harm. On the other hand, the physical and psychological harm between M+G− and M− was still significant after controlling for polydrug and the frequency of using drug, but the social harm is no longer significant after controlling for the frequency. This may reflect that physical and psychological harm are related to the pattern of drug use (i.e., using methamphetamine but not GHB/GBL); however, social harm, which has a wider impact, may be partly explained by the frequency of using a drug rather than the type of drug use or polydrug. Although one study showed that polydrug and methamphetamine use were both associated with high risk of HIV infection and condomless sex [37], few studies have simultaneously compared the relationship between the type of drug use, polydrug, and frequency of using drug and different types of harm.

Research has documented an association between the use of methamphetamine or GHB/GBL and condomless sex with nonsteady partners [38, 39]. In accordance with that, our study showed a significantly higher likelihood of having at least ten sexual partners, inconsistent condom use, having a positive HIV diagnosis, and a diagnosis with at least one STI in the last 12 months among those who used both methamphetamine and GHB/GBL compared to those who used methamphetamine without GHB/GBL, or those who engaged in chemsex without methamphetamine. The reasons behind such an association are likely multifaceted but could conceivably relate to issues of drug dependence [40], or indicate the presence of personality traits that promote sensation seeking [41, 42]. Individuals with sensation-seeking traits may be more prone to engage in high-risk sexual behaviours or to attend chemsex parties [43]. However, we urge caution in interpreting this study, as the evidence is predominantly based on cross-sectional methods, which may not establish causal relationships and could be influenced by undetected confounders. Besides, the effect of disinhibition by GHB/GBL was associated with potentially higher risk sexual behavior [44], which might explain why those who use methamphetamine with GHB/GBL reported higher proportions of more sexual partners, having the diagnosis of HIV positive or at least one STI in the last 12 months than those who use methamphetamine without GHB/GBL.

There were some limitations in this study. First, participants in this study were recruited through one specific popular app used by GBMSM in Taiwan. This might potentially lead to missing those who engaged in chemsex on other apps or channels. Second, our study sample included very few people of M−G+; however, this might reflect the actual situation of the Taiwanese GBMSM chemsex population. Since we merged M−G+ and M−G− into the M− group, it was hard to detect the distinct profile of harm experiences and mental health of those in the M−G+ group. Future studies may make extra effort to recruit more M−G+ users. Third, there might still exist some confounding variables that were not assessed in this study, such as participants' specific psychiatric diagnosis, which could affect their profiles on harm experiences and mental health. Fourth, we did not know for sure whether men were using the mixture of GHB and methamphetamine on the occasion that they experienced harms after the chemsex session. Future study should collect information at the event level and context of the chemsex. For example, a recent study found that in a cohort of young sexual minority men, same-day use of methamphetamine use was most commonly occurred with cannabis and GHB [45].

Research studies have rarely discussed the social harm following a chemsex session. Most studies about chemsex-related social harms have taken a relatively general, sociological, or macroscopic perspective and focused on the social stigma or damage to relationships [4, 46, 47]. Our findings further provide evidence that the harm experienced after a chemsex session is associated with impaired functioning in daily living (such as spending too much time and money on chemsex), social interaction (such as missing dates or appointments), or occupation (such as contributing to unemployment). Harm reduction should aim to assist those who engage in chemsex, and who are facing these kinds of challenging experiences, to manage functions of daily living as well as social interaction, or occupation-related concerns. Experiences within these three dimensions could be used as a checklist of behavioural indicators of chemsex to screen those at risk of potential harm. This behavioural checklist could also assist clinicians in monitoring an individual’s drug use so that help to refer or briefly intervene in their drug use in clinical practice can be delivered. Furthermore, we can enhance support services in the community for individuals experiencing social harm from chemsex and lacking sufficient social support. This could begin by creating a support network within existing clinical services or related non-governmental organizations, allowing individuals to receive tailored harm mitigation services from social workers or frontline healthcare providers. By integrating these resources, the possibility of building a community focused on harm mitigation can be increased.

## Conclusions

Our study revealed that the profile of harm and mental health varied among groups of GBMSM who engaged in chemsex using different combinations of drugs. Typologies of drug use and mixed usage may be key factors differentiating the experience of harm and the interventions that are required to support GBMSM towards safer drug use.

## Data Availability

All the associated data are available on request from authors.

## Abbreviations

GHB/GBL: Gamma-hydroxybutyric acid/gamma-butyrolactone
GBMSM: Gay, bisexual, and other men-who-have-sex-with-men
HIV: Human immunodeficiency virus
STIs: Sexually transmitted infections
PrEP: Pre-exposure prophylaxis
CNS: Central nervous system
COMeT: The Chemsex Online Survey for Men who Have Sex with Men in Taiwan
MDMA: 3,4-Methylenedioxymethamphetamine
ED: Erectile dysfunction
5-meo: 5-Methoxy-diisopropyltryptamine
BSRS-5: 5-Item Brief Symptom Rating Scale
